# Prevalence and factors associated with self-reported HIV testing among adolescent girls and young women in Rwanda: evidence from 2019/20 Rwanda Demographic and Health Survey

**DOI:** 10.1186/s12889-022-13679-8

**Published:** 2022-07-01

**Authors:** Alfred Musekiwa, Patricia Silinda, Assanatou Bamogo, Halima S. Twabi, Mohanad Mohammed, Jesca Mercy Batidzirai, Zvifadzo Matsena Zingoni, Geoffrey Chiyuzga Singini, Maureen Moyo, Nobuhle Nokubonga Mchunu, Theodora Ijeoma Ekwomadu, Portia Nevhungoni, Innocent Maposa

**Affiliations:** 1 School of Health Systems and Public Health, Faculty of Health Sciences, University of Pretoria, Pretoria, South Africa; 2 Aurum Institute, Johannesburg, South Africa; 3 Department of International Health, Bloomberg School of Public Health, Johns Hopkins University, Baltimore, MD USA; 4 Department of Mathematical Sciences, University of Malawi, P.O. Box 280, Zomba, Malawi; 5 School of Mathematics, Statistics, and Computer Science, University of KwaZulu-Natal, Pietermaritzburg, South Africa; 6 Faculty of Mathematical and Computer sciences, University of Gezira, Wad Madani, Sudan; 7 Division of Epidemiology & Biostatistics, School of Public Health, Faculty of Health Sciences, University of the Witwatersrand, Johannesburg, South Africa; 8 Biostatistics Research Unit, South African Medical Research Council, Durban, South Africa; 9 Centre for the AIDS Programme of Research in South Africa (CAPRISA), University of KwaZulu-Natal, 719 Umbilo Road, Durban, 4041 South Africa; 10 Department of Biological Sciences, Faculty of Natural and Agricultural Sciences, North-West University, Mmabatho, 2735 South Africa; 11 Biostatistics Research Unit, South African Medical Research Council, Pretoria, South Africa

**Keywords:** AGYW, DHS, HIV testing, Rwanda

## Abstract

**Background:**

HIV/AIDS remains a major public health problem globally. The majority of people living with HIV are from Sub-Saharan Africa, particularly adolescent girls and young women (AGYW) aged 15-24 years. HIV testing is crucial as it is the gateway to HIV prevention, treatment, and care; therefore this study determined the prevalence and factors associated with self-reported HIV testing among AGYW in Rwanda.

**Methods:**

We conducted secondary data analysis on the AGYW using data extracted from the nationally representative population-based 2019/2020 cross-sectional Rwanda Demographic and Health Survey (DHS). We described the characteristics of study participants and determined the prevalence of HIV testing and associated factors using the multivariable logistic regression model. We adjusted all our analyses for unequal sampling probabilities using survey weights.

**Results:**

There were a total of 5,732 AGYW, with the majority (57%) aged 15-19 years, 83% were not living with a man, 80% were from rural areas, 29% were from the East region, and 20% had a history of pregnancy. Self-reported HIV testing prevalence was 55.4% (95%CI: 53.7 to 57.0%). The odds of ever having an HIV test were significantly higher for those aged 20-24 years (aOR 2.87, 95%CI: 2.44 to 3.37); with higher education (aOR 2.41, 95%CI:1.48 to 3.93); who were rich (aOR 2.06, 95%CI:1.57 to 2.70); with access to at least one media (aOR 1.64, 95%CI: 1.14 to 2.37); who had ever been pregnant (aOR 16.12, 95%CI: 9.60 to 27.07); who ever had sex (aOR 2.40, 95%CI: 1.96 to 2.95); and those who had comprehensive HIV knowledge (aOR 1.34, 95%CI: 1.17 to 1.54).

**Conclusions:**

We report an unmet need for HIV testing among AGYW in Rwanda. We recommend a combination of strategies to optimize access to HIV testing services, especially among the 15-19 years adolescent girls, including facility-based testing, school and community outreach, awareness campaigns on HIV testing, and home-based testing through HIV self-testing.

## Introduction

According to the 2021 UNAIDS report, there were 37.7 million people globally living with HIV (PLHIV) globally in 2020, 36.0 million of whom were adults and 1.7 million children aged 0–14 years, while 53% of all PLHIV were women and girls [1]. Globally, adolescent girls and young women (AGYW), aged 15–24 years, have 60% higher HIV infection compared to adolescent boys and young men of the same age [2]. Furthermore, around 1.5 million people were newly infected with HIV and women and girls accounted for 50% of these new infections. Every week, around 5,000 AGYW become infected with HIV [1].

In Sub-Saharan Africa, AGYW represent 10% of the total population but accounts for about 25% of all HIV infections [3]. Additionally, six in seven new HIV infections among adolescents aged 15– 19 years are among girls [1]. In Eastern and Southern Africa, there were 2.4 HIV infections among AGYW for every one infection among boys and young men of the same age [4]. Adolescent girls and young women remain at a higher risk of HIV infection.

HIV counselling and testing services are essential for HIV prevention, treatment, care, and support [5]. In 2016, the United Nations (UN) declared to end AIDS at the end of 2020 [6]. The declaration endorsed the 90–90-90 targets, requiring at least 90% of PLHIV would know their status, 90% of these would be on antiretroviral treatment (ART), and 90% of those on ART would achieve viral load suppression (VLS) [6]. Increasing access to and uptake of HIV testing is critical to achieving the first and the two corresponding targets [7]. The World Health Organization (WHO) also recommends annual testing in high HIV prevalent settings [7, 8].

Rwanda faces an HIV epidemic with an estimated prevalence in the population aged 15–49 years of approximately 3% in 2010 [9]. The prevalence of HIV among adolescent girls and young women in Rwanda is 3.7% [9]. HIV prevalence discrepancy by gender is particularly marked among young people, where infection among young women of ages 20–24 is five times higher than in men of the same age [10, 11]. The country has made extensive gains in HIV testing and counselling (HTC) as its national strategic plan (NSP) reports an 83.8% rate of self-reported HIV testing among HIV-positive adults aged 15–64 years, specifically 85.6% of HIV-positive women and 80.4% of HIV-positive men [9], thereby showing higher HIV testing among women than men.

Numerous studies have documented factors associated with HIV testing, such as gender, age, education level, pregnancy, geographical location, marital status, and the number of sexual partners [5, 12, 13]. Studies in several countries have found varied HIV testing rates among young women such as in Nigeria (12%), Uganda (26.5%), Kenya (27.7%), and Rwanda (40%) [5]. There remains an unmet need for effective HIV prevention strategies among AGYW compared to their male peers and understanding factors associated with self-reported HIV testing could aid in improving these strategies. This study explored the prevalence and factors associated with self-reported HIV testing among AGYW in Rwanda using 2019/2020 Demographic and Health Survey (DHS) data.

## Methods

### Study design and setting

This is a secondary data analysis of Rwanda’s 2019/2020 DHS data set, a nationally representative population-based cross-sectional survey. The DHS is carried out in all the regions of Rwanda, including rural and urban areas. In Rwanda, the prevention of mother-to-child transmission of HIV (PMTCT) and anti-retroviral therapy (ART) coverage was 96% in 2016 [14]. By 2017, 98% of the health facilities in the country were already offering HIV care and treatment services [14]. Morbidity and mortality among people living with HIV have reduced in recent years due to the coverage of ART, early initiation, and improvements in diagnosis and treatment of opportunistic infections. Rwanda also commenced initiatives targeting adolescents to reduce HIV infection rates, promote changes in programmes and policy as well as create platforms that allow AGYW to have equal access to Sexual and Reproductive Health and Rights (SRHR) services.

### Study population and sampling

The study population was the adolescent girls and young women (AGYW), aged 15–24 years, who lived in Rwanda at the time of the survey. In the survey, stratified, two-stage cluster random sampling of households was done. The 2012 Rwanda population census was used as a sampling frame. The 2019/2020 survey had a household sample size of 13,650 and all eligible members of the selected households who consented to participate were included in the survey. Further details on sampling are available in the 2019/2020 Rwanda DHS report [15]. Among the 14,634 women interviewed for the 2019/2020 Rwanda DHS, 5,732 were qualified to be included in this study.

### Measurements

#### Study outcome

The main outcome of interest was self-reported testing for HIV defined as whether the respondent had ever been tested for HIV. The participants were asked whether they ever had an HIV test, and the responses were either ‘Yes’, ‘No’, or ‘No response’. We treated the ‘No response’ category as missing, and therefore the outcome variable was binary (Yes, No).

#### Predictor variables

The potential factors associated with HIV testing include age category (15–19 years, 20–24 years), employment (Yes, No), marital status, which was re-categorized as living with a man (Yes, No), highest education (No education/primary, Secondary, Higher), place of residence (Urban, Rural), region (Kigali, South, West, North, East), and wealth index (Poorest, Poorer, Middle, Richer, Richest). The exposure to media variable was generated from responses on the frequency of reading newspapers, watching TV, and listening to the radio. Individuals who responded ‘not at all’ to at least one of the sources of media, were coded as 0 and ‘less than once a week and at least once a week’ was given a code of 1. The same applied to watching TV or listening to the radio or reading a newspaper. The final media exposure variable was coded as 0 for all those who said ‘not at all’ for any sources of media and 1 for ‘access to at least newspaper or TV or radio’. Knowledge of mother to child transmission of HIV was coded as ‘Yes’ if a woman responded to knowing HIV transmission through birth, delivery and breastfeeding and 0 if responded ‘No’ to these questions. Comprehensive knowledge of HIV was measured as ‘Yes’ to the following set of questions: reduce risk of getting HIV: always use condoms during sex; reduce risk of getting HIV: have 1 sex partner only who has no other partners; a healthy-looking person can have HIV, and was measured as ‘No’ to the following questions: can get HIV from mosquito bites, can get HIV by witchcraft or supernatural means; would be afraid to get HIV from contact with saliva from an infected person. Similarly, HIV discriminatory attitude was measured as ‘No’ to the following: children with HIV should be allowed to attend school with children without HIV; would buy vegetables from a vendor with HIV; and was measured as ‘Yes’ to the following questions: people hesitate to take HIV test because of the reaction of other people if positive; would be ashamed if someone in the family had HIV. Participants were also asked whether they had been pregnant in the past 24 months (Yes, No). Participants were also asked whether they experienced any form of gender-based violence (GBV); this was measured by saying ‘Yes’ to any one or more of the following: being humiliated; threatened; insulted; pushed; slapped; punched with a fist or hit with something; kicked, burned; threatened or attacked with a knife, twisted; physically forced to have sexual intercourse; physically forced to have any other sexual act, or performed any sexual acts they did not want to.

#### Data management and statistical analysis

The data set is accessible via The DHS Program website (https://dhsprogram.com/data/available-datasets.cfm). We used descriptive statistics to summarize the characteristics of participants and expressed categorical variables as frequencies and percentages with 95% confidence intervals (CI). We carried out a univariate analysis of each predictor variable against the outcome of HIV testing and entered all variables with a *p *- value < 0.1 into the multivariable logistic regression model at the same time. We presented results as crude odds ratios (OR) and adjusted odds ratios (aOR), with corresponding 95% confidence intervals (CI) and p-values. We used a two-tailed *p* < 0.05 to indicate statistical significance. All statistical analyses were conducted using STATA version 14 with “svy” commands to account for unequal sampling probabilities due to the complex survey design. We also used the “svyset” option of “single unit (certainty)” to avoid the model not converging where the numbers were small.

#### Ethics considerations

We sought permission to use DHS data from the DHS program via their website and agreed to all standards and laws applicable in accessing and utilizing DHS data. The Rwandan DHS was ethically approved by the Rwandan Health Research Committee, Institutional Review Board of ICF Macro, and the Centres for Disease Control and Prevention (CDC) in Atlanta, GA, USA, and IRB [15].

## Results

### Cohort description

From the 14,634 women aged 15–49 years included in the 2019/2020 Rwanda DHS, we excluded 8,902 who were aged outside the age group of 15 to 24 years and remained with 5,732 AGYW whom we analysed in this study.

### Characteristics of study participants

The majority of the AGYW (57.4%) were adolescent girls (15–19 years). Most AGYW were not living with a man (83.4%), 79.9% were from rural areas, 28.5% were from the Eastern region, 20.3% from the Southern region, 21.4% from the Western region and 14.4% from the Kigali region. In terms of their education, 49.7% had completed primary education with a similar number having completed secondary education (46.7%), and very few had no education (1.4%). In terms of employment status, 55.4% of the AGYW were either currently employed or had been employed before. In terms of wealth status, 25.4% of the AGYW were from the richest households. Less than 4% had access to at least one of the three communication strategies (newspaper, television or radio). With regards to pregnancy, 20% of the AGYW had been pregnant in the past 24 months. In terms of HIV knowledge, 53.5% had comprehensive HIV knowledge, 66.3% had adequate knowledge of mother-to-child transmission of HIV, and 34.4% did not have HIV discriminatory attitudes. For HIV risky sexual behaviour, 36.9% ever had sex and from these; 9.2% had received cash or gifts in return for sex, 34.1% had two or more sex partners, and 20.2% used a condom during their last sex. Regarding gender-based violence (GBV), 37.6% reported having experienced it. (Table 1).

**Table 1:** Characteristics of adolescent girls and young women, aged 15–24 years, interviewed in the 2019/20 Rwanda DHS (*N* = 5,732)

Characteristics	N (%)^&^	95%CI
**Socio-demographic**		
**Age group, in years**		
15–19	3308 (57.4)	56.0—58.9
20–24	2424 (42.6)	41.1–44.0
**Living with a man**		
No	4828 (83.4)	82.3–84.5
Yes	904 (16.6)	15.5–17.7
**Residence**		
Rural	4305 (79.9)	78.5–81.2
Urban	1427 (20.1)	18.8–21.5
**Region**		
Kigali	750 (14.4)	13.2–15.4
South	1327 (20.3)	18.9–21.7
West	1275 (21.4)	22.0 -22.9
North	903 (15.5)	14.4—16.7
East	1477 (28.5)	26.8—30.3
**Highest education**		
None	76 (1.4)	1.0—1.8
Primary	2830 (49.7)	47.8—51.6
Secondary	2703 (46.7)	44.9—48.5
Higher	123 (2.2)	1.7—2.9
**Employed in the past 12 months**		
No	2563 (44.7)	43.1–46.3
Yes	3169 (55.3)	53.7–56.9
**Wealth index**		
Poorest	952 (16.1)	14.7–17.7
Poorer	1058 (18.9)	17.5–20.3
Middle	1064 (18.8)	17.4–20.2
Richer	1135 (20.4)	19.0–21.9
Richest	1523 (25.8)	23.7–28.0
**Communication strategies**		
**Accesses at least one of the three media at least once a week**		
No	1631 (28.2)	26.6 – 29.9
Yes	4101 (72.8)	70.1—73.4
**Pregnancy Outcome**		
**Ever been pregnant in the past 24 months**		
No	4625 (80)	78.8—81.1
Yes	1107 (20)	18.9—21.2
**HIV Knowledge**		
**Comprehensive HIV knowledge**		
No	3080 (53.5)	51.9–55.1
Yes	2652 (46.5)	44.9–48.1
**HIV discriminatory attitudes**		
Yes	3763 (65.6)	64.1–67.1
No	1969 (34.4)	32.9–35.9
**MTCT knowledge**		
No	1935 (33.7)	32.3–35.2
Yes	3797 (66.3)	64.8–67.7
**HIV sexual risk factors**		
**Ever had sex**		
No	3665 (63.1)	61.7—64.5
Yes	2067 (36.9)	35.5—38.3
	**n (%)**^**&**^	
Cash in return for sex*		
No	560 (90.8)	87.2—93.5
Yes	55 (9.2)	6.5—12.8
Multiple sex partners*		
1 Partner	1368 (65.9)	63.6—68.1
2 Partners	460 (22.3)	20.5—24.1
3 + Partners	238 (11.8)	10.3—13.6
Condom use during last sex*		
No	1093 (79.8)	77.3—82.1
Yes	297 (20.2)	17.9—22.7
Experienced gender-based violence**		
No	135 (62.4)	55.4—68.9
Yes	84 (37.6)	31.1—44.6

^&^_—Percentages are weighted. *The question was only asked from those who had sex, and those with missing data were not included. n—reduced sample size. **Fewer participants answered these questions, *CI*=Confidence Interval, *MTCT*=Mother-To-Child-Transmission of HIV. N of the missing values in the cash in return for sex is 5,117, multiple sex partners is 3,666, condom use during last sex is 4,342, and experienced gender-based violence is 5,513_

### Self-reported HIV testing and associated risk factors among all participants (*N* = 5732)

Table 2 shows the estimates of the factors associated with self-reported HIV testing among AGYW aged 15–24 years. Self-reported HIV testing was 55.4% (95%CI: 53.7 to 57.0%).

**Table 2:** Factors associated with self-reported HIV testing among 15–24 years adolescent girls and young women interviewed in the 2019/20 Rwanda DHS (*N* = 5,732)

	**Univariate analysis**				**Multivariable/Adjusted analysis**		
**Characteristics**	**%HIV Testing**	**Crude OR**	**95%CI**	***P*-value**	**Adjusted OR**	**95%CI**	***P*-value**
**Socio-demographic**							
Age group, in years							
15–19	36.5	1.00			1.00		
20–24	80.9	7.38	6.41–8.49	< 0.001*	2.85	2.42–3.34	< 0.001*
Living with a man							
No	47.4	1.00			1.00		
Yes	95.5	23.77	17.18–32.89	< 0.001*	2.33	1.50–3.61	< 0.001*
Residence							
Rural	52.9	1.00			1.00		
Urban	65.3	1.68	1.42–1.98	< 0.001*	1.27	1.101–1.61	0.045*
Region							
Kigali	65.6	1.72	1.36–2.17	< 0.001*	1.29	0.95—1.74	0.102
South	52.7	1.004	0.84–1.20	0.961	1.17	0.92—1.49	0.208
West	53.0	1.02	0.83–1.25	0.864	1.20	0.92—1.55	0.176
North	57.9	1.24	1.004–1.54	0.046*	1.50	1.15—1.96	0.003*
East	52.6	1.00			1.00		
Highest education							
None/Primary	49.6	1.00			1.00		
Secondary	60.7	1.57	1.40–1.77	< 0.001*	2.24	1.91–2.63	< 0.001*
Higher	77.6	3.53	2.21–5.64	< 0.001*	2.44	1.50–3.98	< 0.001*
Employed in the past 12 months							
No	46	1.00			1.00		
Yes	63	1.99	1.76–2.26	< 0.001*	1.26	1.09–1.47	0.002*
Wealth index							
Poorest	49.4	1.00			1.00		
Poorer	52.3	1.12	0.93–1.36	0.226	1.42	1.10–1.83	0.007*
Middle	52.8	1.15	0.94–1.39	0.172	1.41	1.08–1.83	0.010*
Richer	59.6	1.51	1.24–1.84	< 0.001*	1.81	1.37–2.39	< 0.001*
Richest	59.9	1.53	1.27–1.85	< 0.001*	1.53	1.15–2.03	< 0.001*
**Communication strategies**							
All media							
No	54.8	1.00			1.00		
Yes	70.9	1.44	1.26–1.65	< 0.001*	1.36	1.14–1.63	0.001*
**Pregnancy**							
Ever been pregnant							
No	44.7	1.00			1.00		
Yes	98	60.65	37.74–97.46	< 0.001*	16.83	10.03–28.27	< 0.001*
**HIV sexual risks**							
Ever had sex							
No	38.8	1.00			1.00		
Yes	83.8	8.19	6.95–9.63	< 0.001*	2.38	1.94–2.92	< 0.001*
**HIV Knowledge**							
Comprehensive HIV knowledge							
No	49.2	1.00			1.00		
Yes	62.5	1.71	1.53–1.92	< 0.001*	1.32	1.15–1.52	< 0.001*
HIV discriminatory attitudes							
No	60.8	1.4	1.24–1.58	< 0.001*	0.33	0.17–0.65	0.001
Yes	52.6	1.00			1.00		
MTCT knowledge							
No	52.5	1.00			1.00		
Yes	56.8	1.19	1.05–1.35	< 0.001*	1.08	0.93–1.25	0.311

^*^—*p*—value < 0.05, 95% CI—95% confidence interval

### Socio-demographic factors

In multivariable analysis, the odds of self-reported HIV testing were higher in the 20–24-year-olds compared to the 15–19 year-olds (aOR 2.85, 95%CI: 2.42 to 3.34, *p* < 0.001). AGYW who were living with a man had higher odds of self-reported HIV testing (aOR 2.33, 95%CI: 1.50 to 3.61, *p* < 0.001). Also, those from urban areas had higher odds of self-reported HIV testing compared to rural areas (aOR 1.27, 95%CI: 1.01 to 1.61, *p* = 0.045). Similarly, those from the Northern region had higher odds of self-reported HIV testing than those from the Eastern region (aOR 1.50, 95%CI: 1.15 to 1.96, *p* = 0.003). Compared to those with either no education or only primary education, those who had secondary education (aOR 2.24, 95%CI: 1.91 to 2.63, *p* < 0.001) and those with higher education (aOR 2.44, 95%CI: 1.50 to 3.98, *p* < 0.001)) had higher odds of self-reported HIV testing. The odds of self-reported HIV testing were higher for the employed AGYW (aOR 1.26, 95%CI: 1.09 to 1.47, *p* = 0.002). In comparison with AGYW from poorest households, those who were from poorer (aOR 1.42, 95%CI: 1.10 to 1.83, *p* = 0.007) or middle (aOR 1.41, 95%CI: 1.08 to 1.83, *p* = 0.010) or richer (aOR 1.81, 95%CI: 1.37 to 2.39, *p* < 0.001) or richest households (aOR 1.53, 95%CI: 1.15 to 2.03, *p* < 0.001) had higher odds of self-reported HIV testing. (Table 2).

### Communication strategies

The odds of self-reported HIV testing were higher among those accessing at least one of the three media exposures (radio or newspaper or TV) at least once a week (aOR 1.36, 95%CI: 1.14 to 1.63, *p* = 0.001). (Table 2).

### History of pregnancy

The odds of self-reported HIV testing were higher among those who had been pregnant in the past 24 months (aOR 16.83, 95%CI: 10.03 to 28.27, *p* < 0.001). The odds ratio and its 95% confidence interval were higher and wider, respectively, due to the very high self-reported HIV testing rate of 98% among pregnant women. (Table 2).

### HIV sexual risk behaviours

The odds of self-reported HIV testing were higher among those who ever had sex (aOR 2.38, 95%CI: 1.94 to 2.92, *p* < 0.001). (Table 2).

### HIV knowledge

The odds of self-reported HIV testing were higher among those who had comprehensive HIV knowledge (aOR 1.34, 95%CI: 1.15 to 1.52, *p* < 0.001). However, the odds of self-reported HIV testing were lower among those who had discriminatory attitudes toward the HIV infection (aOR 0.33, 95%CI: 0.17 to 0.65, *p* = 0.001).

## Discussion

The objective of this paper was to determine the prevalence and risk factors for self-reported HIV testing among AGYW, aged 15 to 24 years, in Rwanda using the 2019/2020 Demographic and Health Survey (DHS) data. To do this, we analysed data from 5,732 AGYW which gave a prevalence of 55.4% of self-reported HIV testing among Rwandese AGYW. This is lower than the 78% reported in the 2019/2020 DHS report for the 15–49 year age group. This is probably because women aged 15-49 years have a higher proportion of those with a history of pregnancy than the 15–24 years age group. The HIV testing rates among pregnant women are generally higher since the women are tested for HIV during antenatal care. Although the 2019/2020 DHS reports that 96.1% of 15–24-year-old youths know where to get tested, 45% of the 5,732 AGYW still did not get tested; this may suggest that knowledge of where to get tested does not imply that they would actually test for HIV. The proportion of those testing is also lower because the 96.1% includes both males and females. A logistic regression model accounting for survey weights was performed and findings from the adjusted model show that being in the 20–24 year age group, living with a man, urban-dwelling, higher education, employment, economic stability, access to media, history of pregnancy, ever having sex, HIV non-discriminatory attitudes, and having comprehensive HIV knowledge, were significantly associated with self-reported HIV testing.

We found a strong association between pregnancy and self-reported HIV testing. This could mainly be attributable to the wide access to HIV testing among pregnant women attending antenatal clinic (ANC) visits [16]. Several studies have also found this association [13, 16, 17]. It is important to test for HIV when pregnant as this allows for those who test HIV positive to be enrolled for interventions that prevent mother-to-child transmission of HIV and also to initiate ART.

AGYW aged 20–24 years had higher odds of HIV testing compared to those aged 15–19 years. The age group 20–24 has more information on sexual matters and is more likely to go for HIV testing than the 15–19-year-olds. This is in line with other previously published studies from Zambia, South Africa, and Tanzania [13, 18, 19]. The AGYW who lived with a man were more likely to test for HIV and this could be due to their possibly high sexual activities, hence they are at higher risk of HIV and other sexually transmitted diseases, for which they would visit the healthcare facilities and would also be allowed to test for HIV [20]. Other studies have found similar results, for example in South Africa by Jooste et al. 2020 [21] and in Tanzania by Mahande et al. 2019 [22].

AGYW from urban areas had significantly higher odds for HIV testing compared to their counterparts from rural areas. This could be explained by the easy access to sexual and reproductive health and rights (SRHR) and HIV services in primary healthcare facilities in urban areas. In addition, individuals in the urban areas are modernized and may not be influenced by culture or the stigma that follows when one decides to go for HIV testing as in the rural areas [23]. This finding is supported by various other studies in South Africa, Ethiopia, and Nigeria [5, 24, 25]. We also found that compared to those from the Eastern region of Rwanda, the AGYW from Kigali, and the Northern region were significantly more likely to test for HIV. There is an increase in urbanization caused by demographic shifts and decentralization in the Northern Province. It has also been noted that knowledge and attitudes towards HIV are better than at the national level for the Northern Province [23, 26].

As the education level increased, the odds of HIV testing also increased and this is in agreement with previous studies [27–29]. Those with higher education are also most likely to have higher knowledge of HIV, which we have also found to be associated with testing for HIV. Those who were employed were more likely to have tested for HIV, which can be explained by their chance of exposure to massive HIV awareness events at the workplace or around them. We also found that coming from rich households was associated with HIV testing. This is probably because the poor may not afford transport costs to the HIV testing centres even though HIV testing is free in Rwanda. The rich have more access to all forms of media and therefore are likely to have more knowledge on HIV testing. Additionally, the poor may be more preoccupied with meeting their daily needs and HIV testing may not be a priority for them. Other studies (see Jooste et al. 2020 [21], Cemin et al. 2012 [30], Treves-Kagan et al. 2017 [31], Matovu et al. 2013 [32], Ibrahim et al. [24], Molla et al. [33], Menon et al. [34]) also found that wealthy individuals are more likely to be tested for HIV as compared to poor individuals.

Our findings also show that AGYW who had access to at least one media platform at least once a week (newspapers, TV, or radio) were more likely to have tested for HIV. It is most likely that the local HIV/AIDS organizations and the Rwanda Ministry of Health also disseminate HIV information via newspapers, radios, and TVs. This can sensitise the AGYW on the importance of HIV testing and provide information on where to get HIV tested. This has also been found by other studies from Ethiopia [33] and Zambia [34].

Those who ever had sex were more likely to have tested for HIV. This is not surprising as most HIV transmissions happen through sexual intercourse. This can also be tied to inconsistent condom use [13], which is associated with HIV testing. The fear that one may have acquired HIV after having unprotected sex can influence an individual to go for HIV testing.

This study is not without limitations. Since this is a secondary data analysis of a cross-sectional study, we cannot infer a causal relationship between the risk factors and the self-reported HIV testing outcome. In addition, the data set lacked some known risk factors for HIV testing; for instance, religious beliefs can influence the decision for HIV testing. We also could not determine whether some HIV sexual risk behaviours such as inconsistent condom use, multiple sexual partnerships, and inter-generational sex partnerships, were associated with HIV testing because our model could not converge when these were included. Moreover, the data on gender-based violence (GBV) had a lot of missing data. This may be due to the sensitive nature of the subject that could have underestimated the association between intimate partner violence and/or GBV with testing for HIV.

There are also strengths of this study. This study is a secondary data analysis of a recent large sample of 5,732 Rwandese AGYW with data from DHS, which is a rigorously conducted nationally representative population-based cross-sectional survey. All analyses done on the survey data were adjusted using survey weights. 

## Conclusion

In summary, we found an unmet need for HIV testing among adolescent girls and young women in Rwanda based on the 2019/2020 DHS data. We also found that being 20-24 years old, living with a man, urban-dwelling, higher education, employment, economic stability, access to media, history of pregnancy, ever having sex, and having comprehensive HIV knowledge were associated with HIV testing. We, therefore, recommend that the Rwandan Ministry of Health and other stakeholders incorporate our determinants of self-reported HIV testing in creating awareness for HIV testing among youths. We also recommend qualitative studies to further describe the barriers and facilitators of self-reported HIV testing among adolescent girls and young women in Rwanda.

## Data Availability

The dataset generated and analysed during the current study are not publicly available since we received a data access letter from the DHS team https://dhsprogram.com/ specific to our project but are available from the DHS team upon request.

## Abbreviations

AGYW: Adolescent Girls and Young Women
DHS: Demographic and Health Survey
HCT: HIV Counselling and Testing
MOH: Ministry of Health
RDHS: Rwanda Demographic and Health Survey
SRHR: Sexual and Reproductive Health and Rights
WHO: World Health Organization
